# Bridging ecological assembly process and community stability upon bacterial invasions

**DOI:** 10.1093/ismejo/wrae066

**Published:** 2024-04-25

**Authors:** Xipeng Liu, Joana Falcão Salles

**Affiliations:** Microbial Ecology Cluster, Genomics Research in Ecology and Evolution in Nature (GREEN), Groningen Institute for Evolutionary Life Sciences (GELIFES), University of Groningen, 9747 AG Groningen, The Netherlands; Microbial Ecology Cluster, Genomics Research in Ecology and Evolution in Nature (GREEN), Groningen Institute for Evolutionary Life Sciences (GELIFES), University of Groningen, 9747 AG Groningen, The Netherlands

**Keywords:** microbial invasion, pathogen dispersal, resources competition, life strategy, stochasticity, resistance

## Abstract

Understanding the link between microbial community stability and assembly processes is crucial in microbial ecology. Here, we investigated whether the impact of biotic disturbances would depend on the processes controlling community assembly. For that, we performed an experiment using soil microcosms in which microbial communities assembled through different processes were invaded by *Escherichia coli.* We show that the ecological assembly process of the resident community plays a significant role in invader-resident competition, invader survival, and compositional stability of the resident community. Specifically, the resident communities primarily assembled through stochastic processes were more susceptible to invader survival. Besides, *E. coli* invasion acts as a biotic selection pressure, leading to competition between the invader and resident taxa, suppressing the stochasticity in the resident community. Taken together, this study provides empirical evidence for the interpretation of microbial community assemblage on their (potential) ecosystem functions and services, such as the prevention of pathogen establishment and the pathogenic states of soil microbiomes.

Soil microbial communities are critical to various terrestrial ecosystem processes and services, including plant development, disease dispersal, biogeochemical cycles, and climate change regulation [1]. The functions that microbial communities perform are intensively linked to their diversity, structure, and, theoretically, to the processes that regulate the community assembly [2–5]. Species assemblages can be shaped by deterministic (e.g. selection) and stochastic (e.g. drift and dispersal) factors simultaneously, where each factor varies in its relative importance in controlling community composition [6, 7]. Advances in DNA sequencing and statistical tools have allowed us to quantitatively estimate the assembly processes of microbial communities [8–10]. Numerous studies have recently investigated the processes controlling soil microbial community assembly in various habitats—from a historical perspective—across environmental types, climate conditions, and events that are driven by human activities [6, 11, 12]. However, it remains poorly explored whether and how the assembly processes of soil microbial communities influence emergent community properties, such as compositional stability, in response to disturbances.

In this study, we explored whether the impact of biotic disturbances, i.e. the invasion of the bacterial species *Escherichia coli*, would depend on the processes controlling community assembly. We hypothesize that communities driven primarily by stochastic processes would promote invader establishment due to variable interspecies interactions, lessening species’ competition for resources and niche space. We further hypothesized that the invasion’s impact on the resident community would be directly related to the competition triggered by the invader. Moreover, as invasion acts as a biotic selection pressure, it is expected to strengthen the deterministic (selection) process structuring the resident community.

To address our hypotheses, we prepared four resident communities structured by different assembly processes in soil microcosms. This was achieved by introducing fresh soil suspensions to recipient soils, which were allowed to be colonized for 0, 5, 10, and 20 days. The difference in the colonization time mimicked different levels of interspecies interactions controlled by different assembly processes (see Supplementary information for experimental details; Supplementary Fig. S1). The soil bacterial density of resident communities was kept at the highest carrying capacity of soils and at a similar level throughout the experiment. We observed no significant difference in the number of culturable cells among four resident communities before the invasion (measured with plate counting; Supplementary Fig. S3). Using 16S rRNA gene amplicon sequencing (see Supplementary information for experimental details; Supplementary Fig. S2), we revealed that the alpha diversity of resident communities was highest in the community colonized for 0 days (C0) (Supplementary Fig. S4) and that the structures of resident communities were different from each other (Supplementary Fig. S5). We further used the relative importance of stochasticity estimated by the phylogenetic-bin-based null model analysis (iCAMP) [10] to infer the assembly processes of resident communities at the onset of the invasion. Our results showed that the stochasticity of communities increased with the colonization duration and showed larger than 50% after 5, 10, and 20 days (C5, C10, and C20) (Supplementary Fig. S6). Specifically, these four communities were mainly driven by homogenous selection, while the longer colonization duration decreased the relative importance of homogenous selection and promoted homogenizing dispersal and drift (Supplementary Fig. S7).

The microcosms were subsequently subjected to microbial invasion by introducing the invader, *E. coli*, added into four resident communities at 7.5 × 10^8^ CFU in 0.1-ml suspension, achieving an invasion rate of 4.42% in bacterial density. After the invasion, the invaded communities were incubated for an additional 20 days, and the uninvaded treatments were set as the control. After the invasion period, *E. coli* survival and bacterial community structure were measured by counting on antibiotic agar plates and 16S rRNA gene amplicon sequencing, respectively.

Twenty days after the invasion, *E. coli* survival was higher in recipient soil (with lower bacterial density) than in soils where microbial communities had been introduced (C0-C20; Fig. 1A). This indicated that the high-density bacterial communities constrained invader survival. Moreover, we showed that the *E. coli* survival was also significantly different in the four resident communities where the highest survival rate was shown in C20 (Fig. 1A). However, such difference between treatments was not due to the initial bacterial density of resident communities (Supplementary Fig. S8a). Furthermore, prior knowledge that resident communities with higher alpha diversity are less favorable for the establishment of invaders, also known as the biodiversity-invasion relationships [13, 14], did not apply in our case (Supplementary Fig. S8b). Here, we showed that *E. coli* survival was significantly correlated with increased stochasticity in resident communities (Fig. 1B). Moreover, the abundance-weighted ribosomal RNA operons (*rrn*) copy number of bacterial communities indicated that the community shifted from r-to k-strategy as the colonization time increased, which indicates that the community’s preference for labile resources was more intensive in C0 (copiotrophic) and gradually weakened in C5, C10, and C20 (oligotrophic) [15]. Such change was significantly related to the community assembly process (Fig. 1C), implying the substantial relationship between community succession and microbial life strategies, where oligotrophic species (low *rrn* copy numbers, k-strategists) are driven by stochastic processes and more prone to invasion than those dominated by r-strategists. These results suggest the speed at which the resources are consumed by the native communities composed of fast-growing copiotrophic organisms (high *rrn* copy numbers; r-strategists) induces selection, possibly through competitive exclusion, reducing the success of the invasion. However, as succession takes place and labile resources are less available, oligotrophic microorganisms take over. This successional pattern promotes stochasticity due to an increase in the relative importance of drift and *E. coli* survival. Variation partitioning analysis (VPA) suggests that the shift of residents’ life strategy (estimated by *rrn*) and stochasticity in resident communities together contributed to *E. coli* survival (Fig. 1D). Overall, our findings suggest that the competition, e.g. for labile resources and niche space, is the main reason for suppressing *E. coli* survival in soil. Further studies that manipulate soil labile compounds while verifying microbial life strategies and community assembly processes for longer periods are needed to verify our findings.

**Figure 1:**
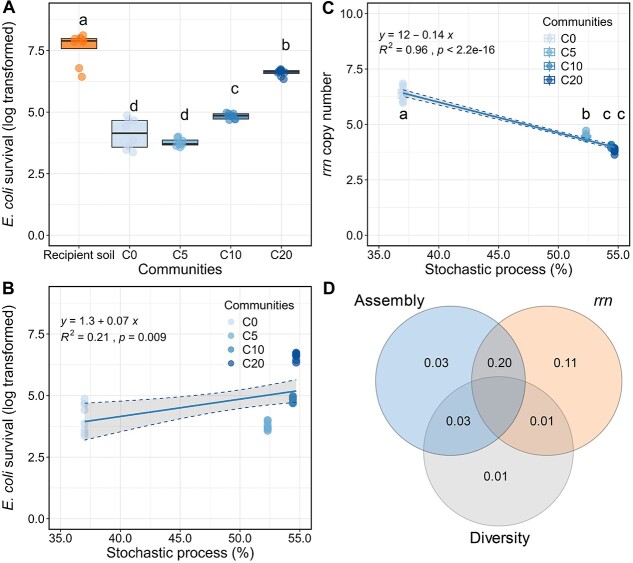
Invader (*E. coli*) survival and its relationship with the assembly process of resident communities at the onset of invasion; (A) the *E. coli* survival in soils where communities colonized with different durations, and the Pearson’s correlation between the assembly process of resident communities and (B) the *E. coli* survival and (C) the *rrn* copy number of resident communities; (D) VPA assessing predictors influencing *E. coli* survival, and this analysis was based on results from treatments C0, C5, C10, and C20; variations explained by three variables were labeled (global model: *P* = .003, degrees of freedom: 3, *N* = 32, residuals = 0.69, values less than 0 were not shown), and assembly, the relative importance of stochasticity of resident communities estimated by iCAMP; *rrn*, the ribosomal RNA operons (*rrn*) copy number of resident communities, representing the potential life strategy (k-/r-strategy) of resident taxa; diversity, the richness of resident communities; different letters indicate significant differences analyzed by ANOVA with post-hoc Tukey HSD test (*P* < .05, *N* = 8).

We assessed the invasion impact on resident community structure by calculating the weighted UniFrac dissimilarity between invaded and uninvaded communities (Fig. 2, Supplementary Figs S9 and S10). We observed that the structure of resident communities changed more in treatments C0 and C5, which are less governed by a stochastic process, than in C10 and C20 (Supplementary Fig. S9a), indicating that survival was aligned with the competition the invader suffered. The *E. coli* survival was also significantly and negatively correlated with the invasion impact (Supplementary Fig. S9b). These results reinforce that the competition between the invader and residents determines the invader’s survival and the corresponding shift in resident community structure. A similar phenomenon has been validated in *Bacillus* invasions [16]. Moreover, we found that more ASVs significantly changed in C0 and C5 (2.24% and 2.48%, respectively) compared with that in C10 and C20 (1.60% and 1.02%, respectively, Supplementary Fig. S11a and b). A well-publicized and recently proven idea in microbial ecology is the competition-relatedness hypothesis which suggests that antagonism is primarily prevalent among phylogenetically and metabolically similar bacterial species [17, 18]. We also found that ASVs with decreased abundance were phylogenetically closer to the genus *Escherichia* than ASVs with increased abundance (Supplementary Fig. S11c, d, and Table S1). Thus, our findings suggest that the competition between the invader and residents is driving the alteration of resident community structure after invasions.

**Figure 2:**
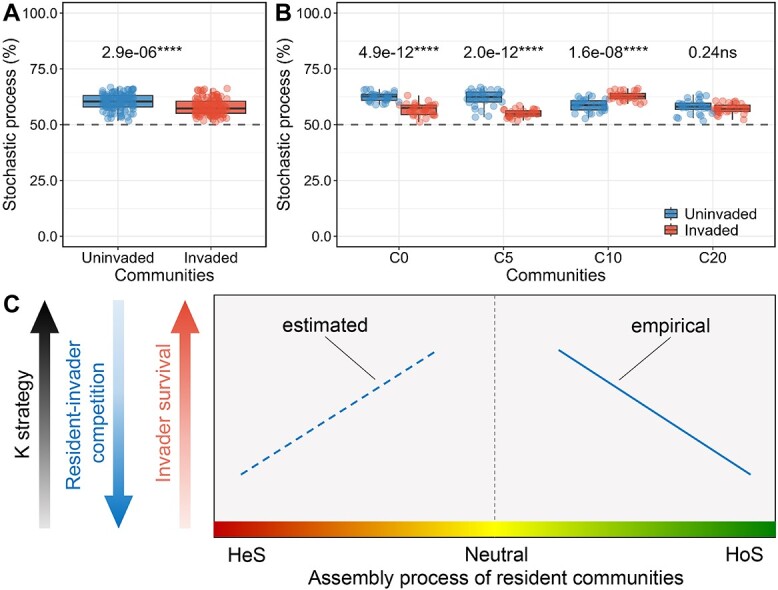
The impact of *E. coli* invasion on the resident community assembly 20 days after invasion, and (A, B) *E. coli* invasion inhibits the stochastic assembly process of the resident community; the *P*-value followed by asterisks represent statistical significance between invaded and uninvaded treatments measured by ANOVA with post-hoc Tukey HSD test; (C) the illustration of the relationship between the assembly process and invader-resident competition, and the resident community tends to adopt the K strategy when the stochastic process increases and homogeneous selection decreases, resulting in weakened competition with invaders, and the solid line represents the empirical relationship summarized by the results from this study, and the dashed line represents the estimated relationship that could be further explored; HeS, HoS, and neutral indicate that the resident community was mainly assembled with heterogenous selection, homogeneous selection, and stochastic processes.

The *E. coli* invasion significantly decreased the relative importance of the stochastic process of invaded communities (Fig. 2A), reflecting the biotic stress imposed on the soil microbial community [19]. Specifically, the decrease in stochasticity was more significant in C0 and C5, than in C10 and 20, which is consistent with the changes in community compositions (Fig. 2B and Supplementary Fig. S11). Upon invasions, the role of *E. coli* invasion on C0 and C5 was strengthening the homogenous selection and decreasing the dispersal limitation and drift (Supplementary Fig. S12). Furthermore, we observed that community assemblage responded differently to *E. coli* invasion in different treatments (e.g. in C10 and C20), suggesting that the impacts may depend on the properties of the resident communities.

In summary, this study bridges the assembly process of soil microbial communities to their competitivity and compositional stability against *E. coli* invasion. Our results suggest that the microbial communities dominated by r-strategists promote homogeneous selection pressure, possibly due to stronger resource competitiveness. This, in turn, has a strong impact on invasion, leading to a lower invader’s survival rate. As resources are consumed during microbial growth and succession, resident communities shift from copio to oligotrophic organisms, and strong homogeneous selection is replaced by drift, leading to a higher survival rate. Future studies should verify whether these patterns hold in long-term experiments, as our study focused on the short-term monitoring of invasion consequences (20 days). Moreover, additional experiments should verify whether the invasion impact we observed at the compositional level also translates into changes in community functionality, e.g. invasion impact on carbon and nutrient cycling, placing the conclusions on the long-term consequences in the context of the functions and services provided by the communities. Finally, translating these findings to natural soil ecosystems has limitations, as soil physiochemical properties and fluctuations in temperature and humidity also influence how communities are formed, making the interpretation of the ecological significance of community assemblage more challenging than in controlled microcosms. Nevertheless, this study is motivating us to unravel the extent to which the assembly process of soil microbial communities can be used to reflect their temporal constancy (variations in community structure) and resource utilization strategy in other contexts, such as invasion occurring in communities driven by heterogenous selection (Fig. 2C) and communities facing other abiotic disturbances. In doing so, a more accurate understanding and prediction of the stability of microbial communities in this rapidly changing world will likely be developed, allowing us to steer ecosystem functions such as tackling the climate change challenge and preventing the spread of pathogens.

## Supplementary Material

Supplementary_information_E_3_corrected_wrae066

## Data Availability

All the raw sequencing data were deposited in the National Center for Biotechnology Information Sequence Read Archive under the accession number PRJNA1005469. R code used for data interpretation, statistical analysis, and figure generation is available at https://doi.org/10.17605/OSF.IO/R9ZW3.
