# Supplementary material for: Bridging ecological assembly process and community stability upon bacterial invasions
Source: ISME J. 2024 Apr 25;18(1):wrae066. doi: 10.1093/ismejo/wrae066 (PMC11159528; doi:10.1093/ismejo/wrae066)
Supplement: Supplementary_information_E_3_corrected_wrae066 [file supplementary_information_e_3_corrected_wrae066.docx]

**Supplementary Information**

**Bridging ecological assembly process and community stability upon bacterial invasions**

Xipeng Liu, Joana Falcão Salles^*^

*Microbial Ecology cluster, Genomics Research in Ecology and Evolution in Nature (GREEN), Groningen Institute for Evolutionary Life Sciences (GELIFES), University of Groningen, 9747 AG Groningen, The Netherlands*

**^*^Corresponding authors**: Joana Falcão Salles ([j.falcao.salles@rug.nl](mailto:j.falcao.salles@rug.nl))

The supplementary information includes Supplementary Methods, Figure S1-12, and Table S1.

**Supplementary Methods**

*Microcosm setup*

Four resident communities were created by introducing a fresh soil suspension into a recipient soil. The recipient soil in this study was prepared by collecting fresh soil from a potato field (sandy loam, pH 4.75) in Friesland, the Netherlands, and gamma irradiating it (50 kGy) [1]. By counting the bacteria on R2A agar plates, the bacterial density of this recipient soil was 4.2 × 10^5^ CFU/g dry soil. The 80 g recipient soil was thoroughly mixed with 240 ml fresh soil suspension (2.3 × 10^10^ CFU/ml, measured by plate counting four days in advance) to obtain the resident soil with a bacterial density of 7.5 × 10^9^ CFU/g dry soil. In doing so, the bacterial density of different communities was expected to be stable without a large extent of increase. The mixed soil was divided into the 24-well plate (DNA free, Corning Costar TC-Treated Multiple Well Plates), and each well contained 3 g of soil representing a replicate. Four plates containing 96 samples were created and incubated at 26 ºC in darkness (Supplementary Figure S1).

*Experimental design*

Each 24-well plate was divided into three blocks, namely the resident community before the invasion, the uninvaded community, and the invaded community, and each block contains eight replicates. Four 24-well plates were used as four treatments in which the resident communities were allowed to colonize for 0, 5, 10, and 20 days before invasions and referred to as C0, C5, C10, and C20, respectively (Supplementary Figure S1). The soil samples of the resident communities were collected before invasion events.

After colonizing for a corresponding period, the invader, *Escherichia coli* O157: H7 (*E. coli*, a non-pathogenic variant of a severe pathogen), was introduced into soils at a 7.5 × 10^8^ CFU density with 0.1 ml suspension to achieve a 4.4% invasion rate. For the uninvaded treatments, 0.1 ml of sterilized water was added simultaneously. The soil samples from invaded and uninvaded treatments were collected 20 days after the invasions. The soil water content on four plates was kept stable by weighing and keeping the same weight by supplementing with sterile water.

*Bacterial density measurements*

Invader survival from samples collected 20 days after invasion was tracked from fresh soil via selective dilution plating on Trypticase Soy Agar (TSA) supplemented with rifampicin (10 μg/ml) and kanamycin (50 μg/ml), both antibiotics to which this strain of *E. coli* harbored resistance. Soil culturable bacterial density was measured via dilution plating on R2A agar added with fungicide Cycloheximide (100 μg/ml).

*Soil DNA extraction, 16S rRNA gene sequencing, and bioinformatic processing*

The genomic DNA was extracted from 100 soil samples (the extra four samples were taken from the recipient soil). In brief, 0.25 g of soil was used to isolate DNA with the DNeasy PowerSoil Pro Kit (QIAGEN, Germany) according to the manufacturer’s instructions. The prokaryotic genomic libraries were prepared and sequenced at the University of Minnesota Genomics Center (UMGC) following the two-step dual-indexing approach [2]. The produced 16S_V4 amplicons that were constructed with primer pair 515F (5’-GTGCCAGCMGCCGCGGTAA-3’) and 806R (5’-GGACTACHVGGGTWTCTAAT-3’) were sequenced by 2x300 bp MiSeq at UMGC to characterize prokaryotic communities.

We used the QIIME2 pipeline (version 2020.8) to process 16S rRNA gene sequencing data. First, the quality control and removal of low-quality regions of the sequences were performed with DADA2 to infer Amplicon Sequence Variants (ASVs). Next, taxonomy was assigned to representative sequences using the Silva 138 Naive Bayes 515F/806R classifier. All ASVs affiliated with archaea, eukaryotes, mitochondria, and chloroplast were removed from the dataset. The pipeline FastTree generated a phylogenetic tree from representative sequences by aligning sequence fragments via the MAFFT program. To make samples comparable, the feature table of each sample was rarefied to a depth of 13800 sequences (Supplementary Figure S2).

*Abundance-weighted averaged rrn copy number of bacterial communities*

The ribosomal RNA operons (*rrn*) copy number of bacterial communities might reflect the community-level trait associated with r/K selection and their life strategies [3]. The mean copy number of ribosomal RNA operons (*rrn*) for each ASV was estimated through the rrnDB database (https://rrndb.umms.med.umich.edu/) based on its closest relatives with known *rrn* copy number [4]. The *rrn* copy number searching was conducted based on the downloaded data set of rrnDBs (rrnDB-5.8_pantaxa_stats_RDP.tsv.zip) modified on 23rd June 2022. The abundance-weighted average of the *rrn* copy number for each bacterial data set was calculated by the mean operon copy number and the corresponding abundance for each ASV [5]. In general, bacterial communities with fewer *rrn* operons tend to adopt K-strategy (K strategist; oligotrophic species) which have lower growth rates and more efficient utilization of recalcitrant resources [3, 6].

*Statistical analyses*

Analyses and visualizations were performed in R. Analysis of variance (ANOVA) was performed with Tukey's HSD test, and the variances were visualized with the “multcompView” package [7]. Pearson’s correlation analyses were used in this study for the (multiple) linear regression fitting, and the significance was tested with the “ggpubr” package [8]. To evaluate the impact of invasion on soil microbial communities, the dissimilarity/similarity values based on weighted UniFrac distance and Bray-Curtis were calculated using the R package “vegan” [9]. The dissimilarity between invaded and uninvaded communities was used to reflect the compositional stability of resident communities upon invasions. Variation partitioning analysis (VPA) was conducted using the *varpart* function in the package “vegan”. A recent reported method for assessing the assembly process based on phylogenetic-bin-based null model analysis was used with the package “iCAMP” [10] (metric: bMPD; null model significance test: confidence; bin size: 24). The relative importance of stochastic processes (homogenizing dispersal, dispersal limitation, and drift) based on iCAMP were obtained. The stochasticity results obtained by iCAMP lie between 0 and 1 (calculated by 1 – Heterogeneous selection – Homogeneous selection), representing the relative importance of the stochastic process of the community, where 1 represents 100% stochasticity. The package “DESeq2” [11] was used to analyze the differential abundance of ASVs between invaded and uninvaded communities. The phylogenetic distance between different ASVs was calculated with the package “ape” [12].

**Supplementary Figures**


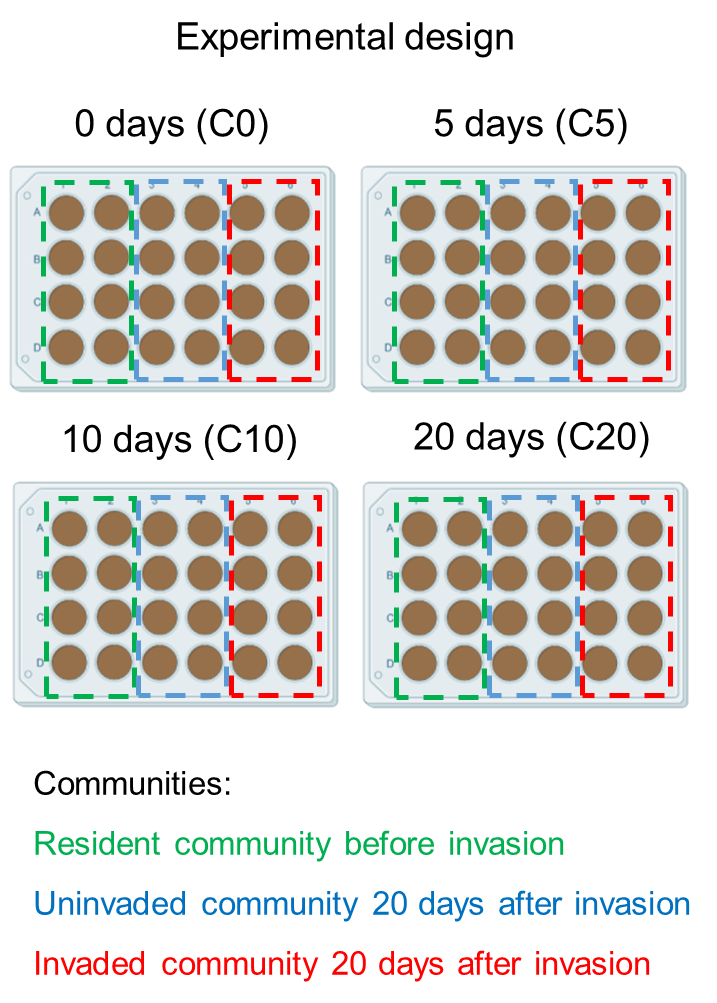


Figure S1. The experimental design. Three gram of soil was distributed into each well. Green, blue, and red represent three communities: resident communities colonized for 0, 5, 10, and 20 days, uninvaded communities after 20 days, and invaded communities 20 days after *E. coli* invasions.


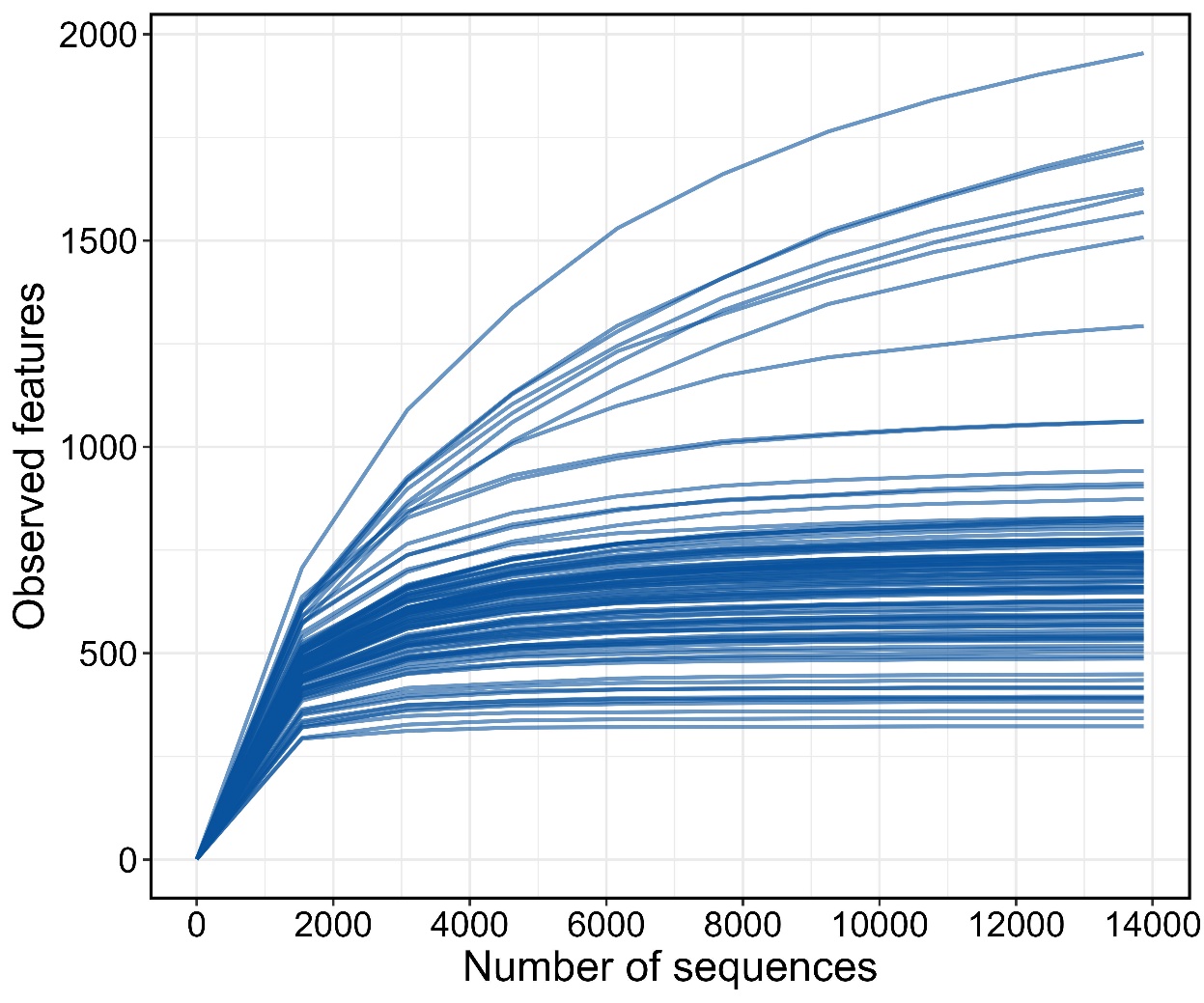


Figure S2. The rarefaction curves of all samples. The feature table of each sample was rarefied to a depth of 13800 sequences.


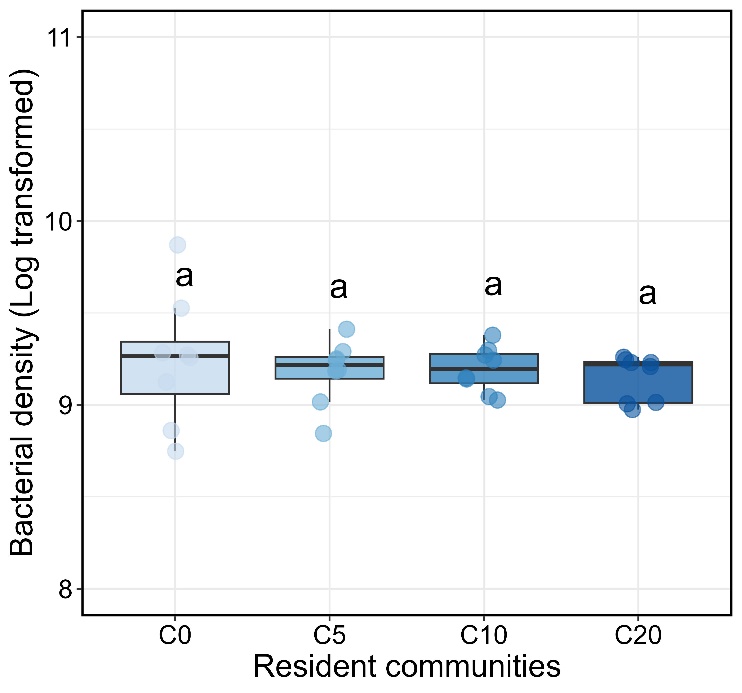


Figure S3. The culturable bacterial density of resident communities before the invasion. Different letters above boxplots indicate significant differences analyzed by ANOVA with post-hoc Tukey HSD test (*p* < 0.05, N=8). The bacterial density of the recipient soil was 4.2 × 10^5^ CFU/g dry soil (the log-transformed value: 5.6).


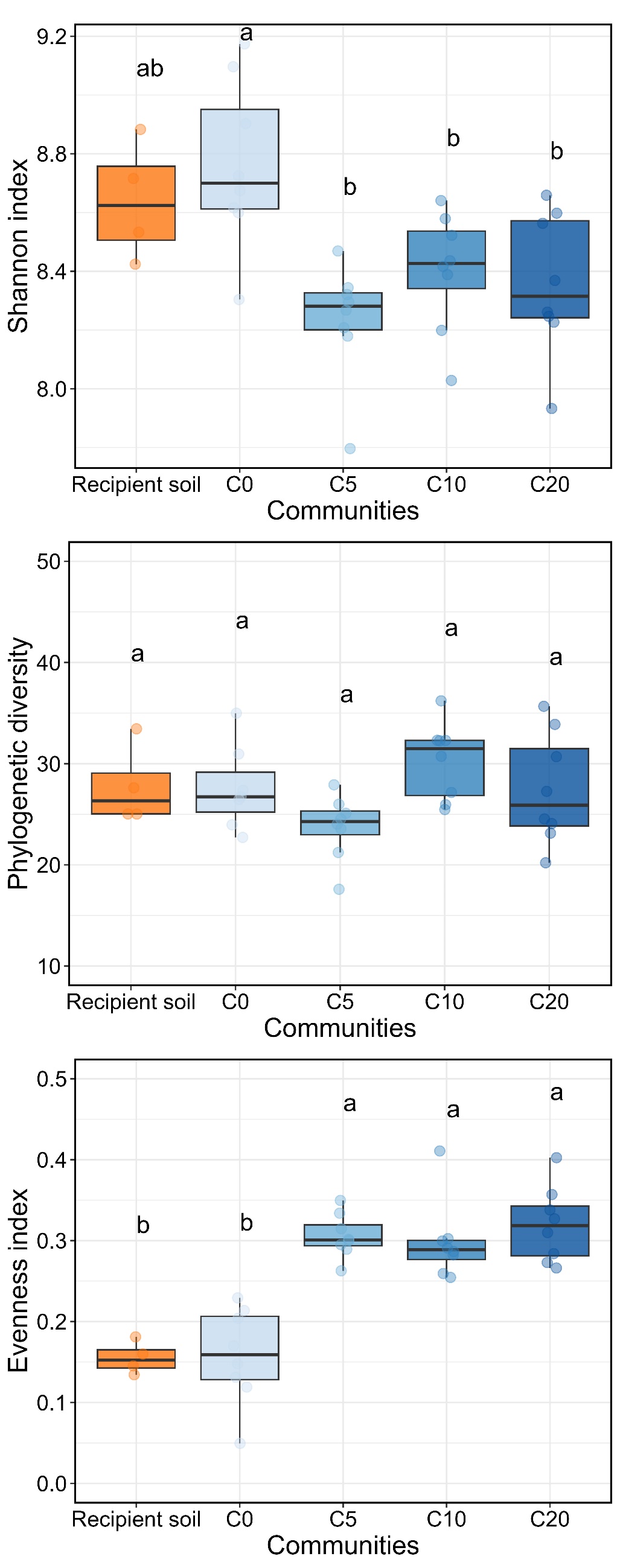


Figure S4. The alpha diversity of resident communities before the invasion. Different letters above boxplots indicate significant differences analyzed by ANOVA with post-hoc Tukey HSD test (*p* < 0.05, N=8).


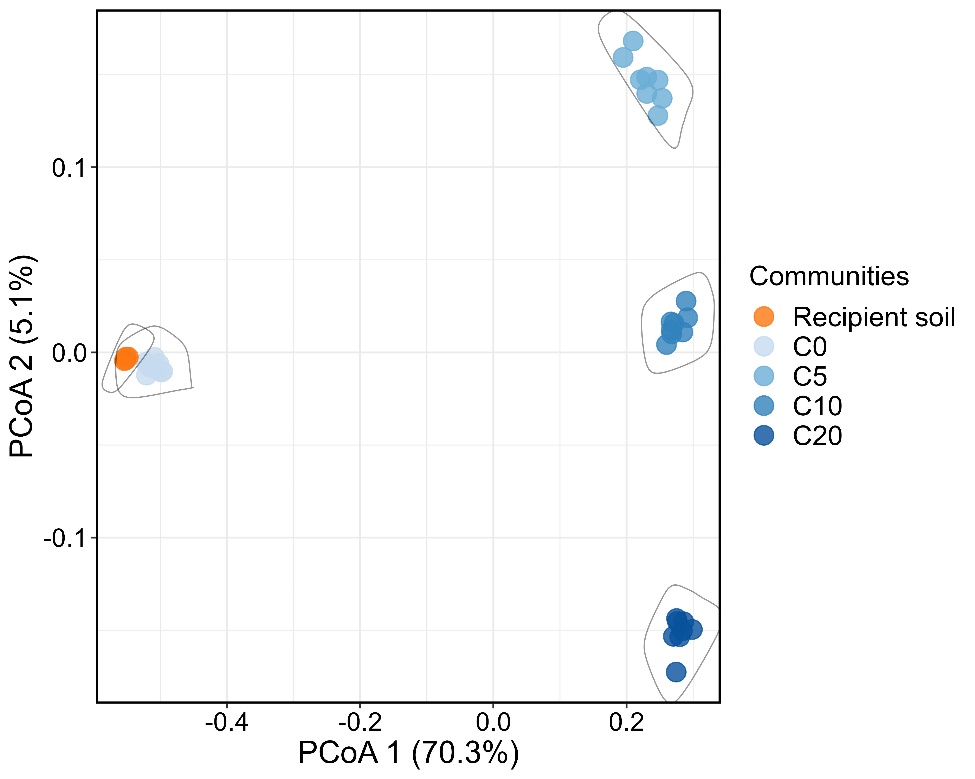


Figure S5. The principal coordinates analysis (PCoA) of resident community structures before the invasion is based on the weighted UniFrac distance.


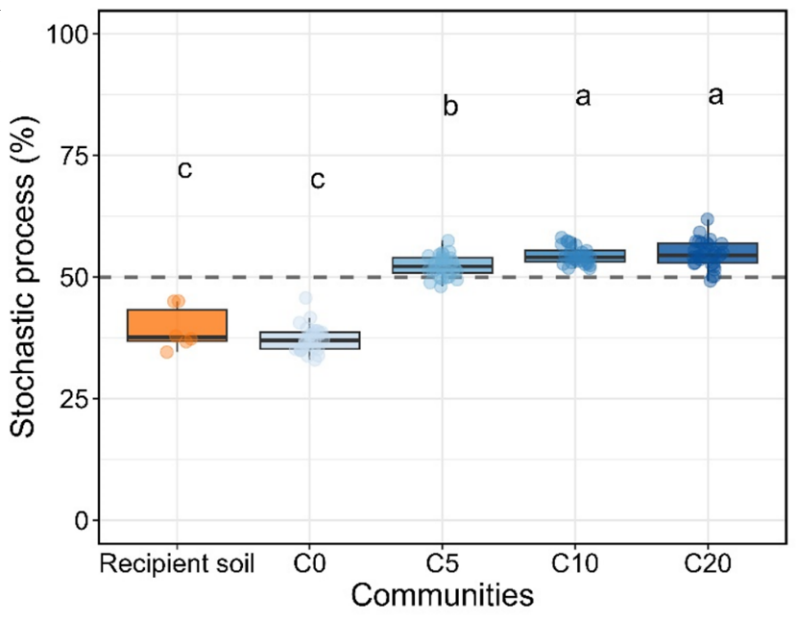


Figure S6. The assembly process of resident communities before the invasion estimated by iCAMP. Different letters above boxplots indicate significant differences analyzed by ANOVA with post-hoc Tukey HSD test (*p* < 0.05).


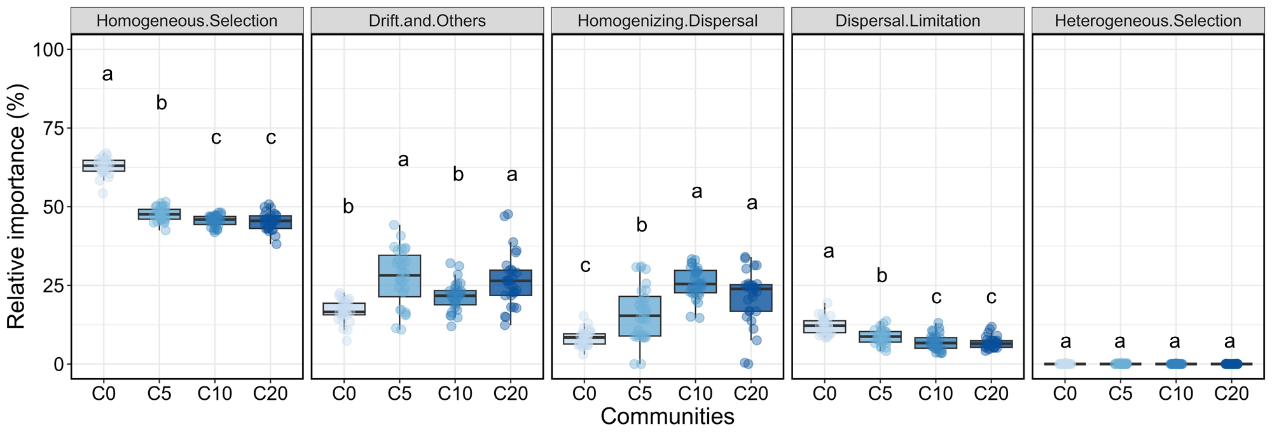


Figure S7. The assembly process of resident communities before the invasion estimated by iCAMP. Different letters above boxplots indicate significant differences analyzed by ANOVA with post-hoc Tukey HSD test (*p* < 0.05).


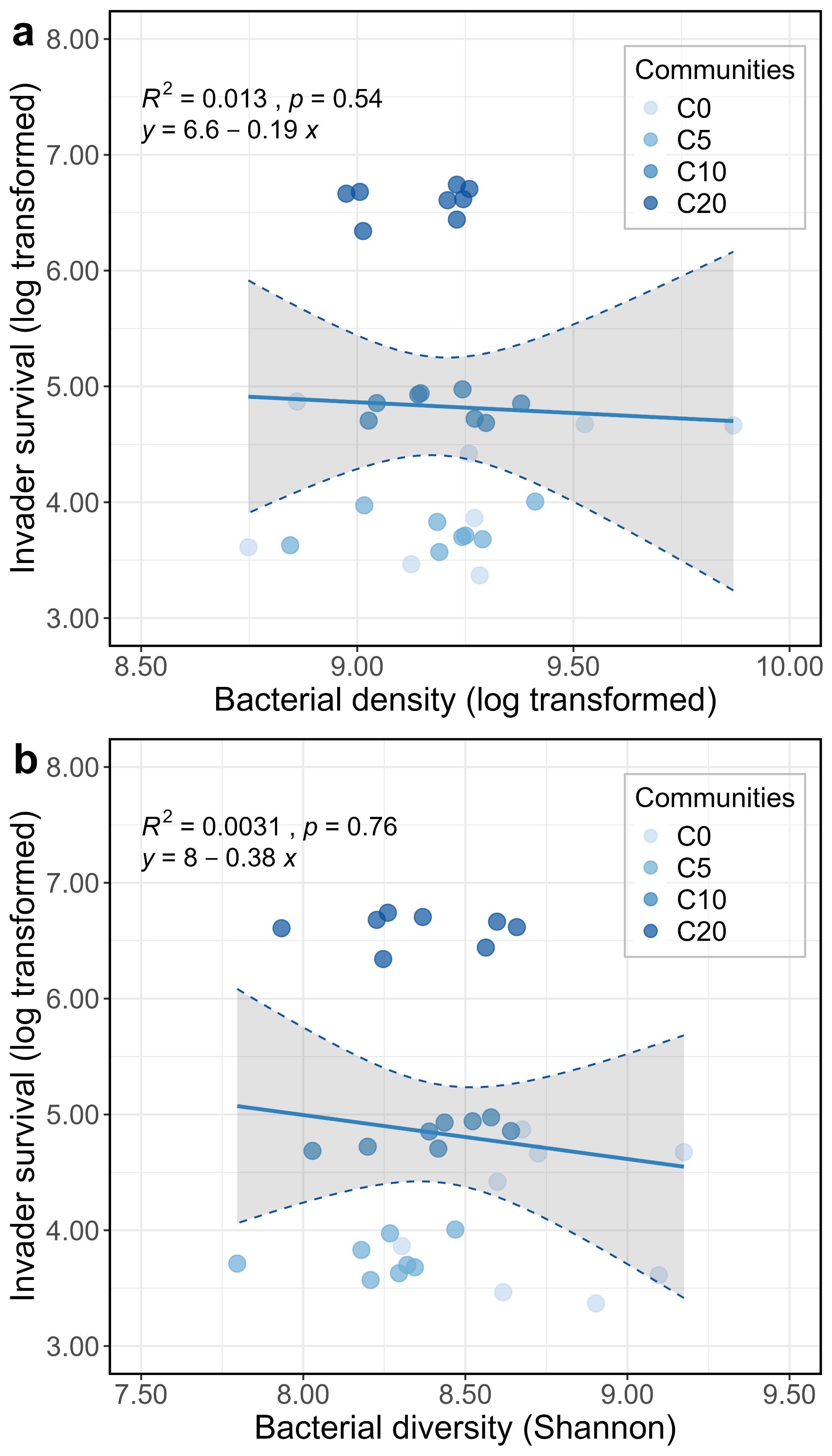


Figure S8. The Pearson’s correlation between the initial bacterial density (a) and Shannon diversity (b) of resident communities and *E. coli* survival after the 20-day invasion (N=32).


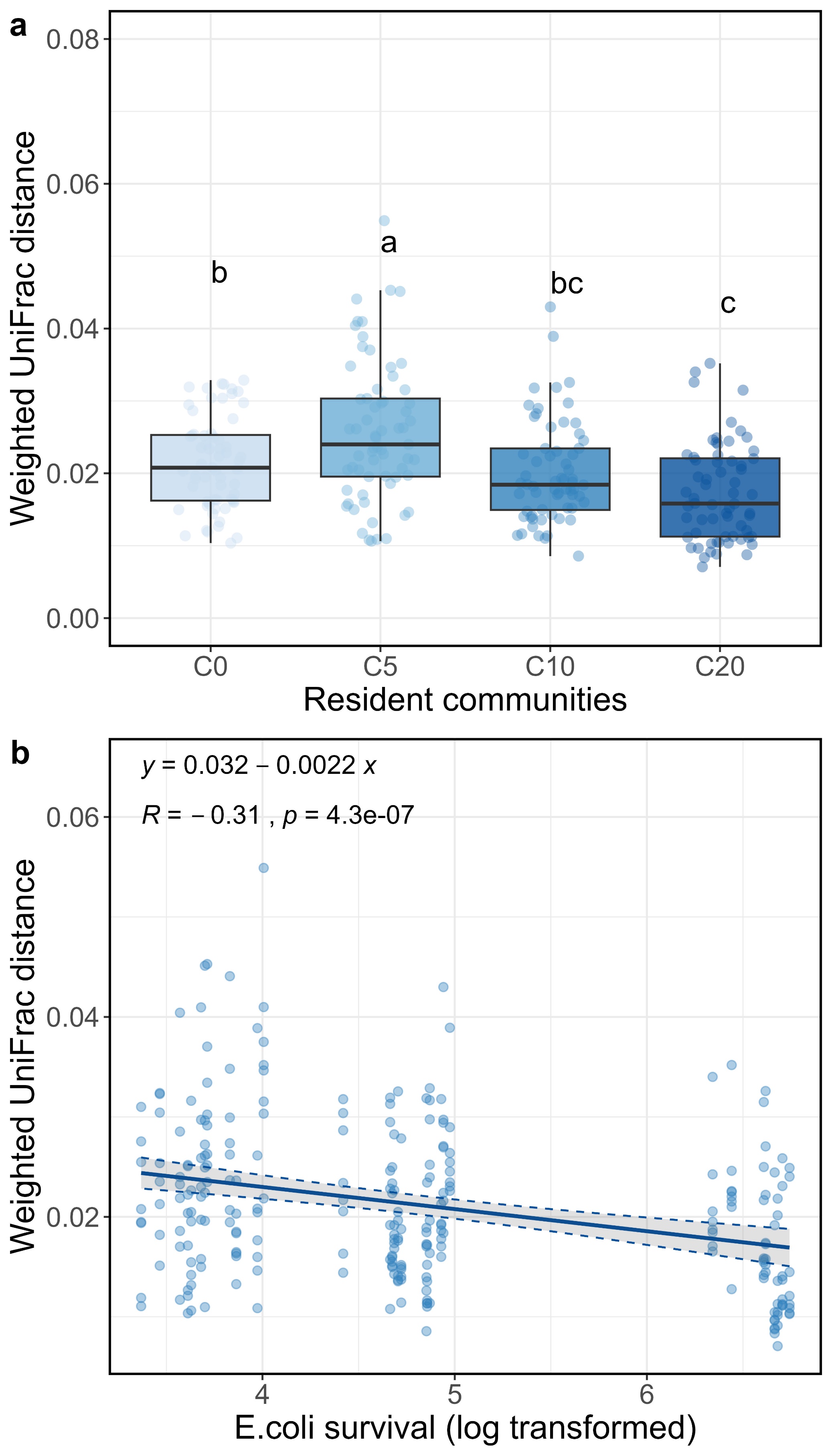


Figure S9. (a) The invasion impact on resident community structure after 20 days. The impact was assessed with weighted UniFrac distance between invaded and uninvaded communities. The dissimilarity between invaded and uninvaded communities was used to reflect the compositional stability of resident communities upon invasions. Different letters above boxplots indicate significant differences analyzed by ANOVA with post-hoc Tukey HSD test (*p* < 0.05). (b) The Pearson’s correlation between invasion impacts and log-transformed values of *E. coli* survival after 20 days.


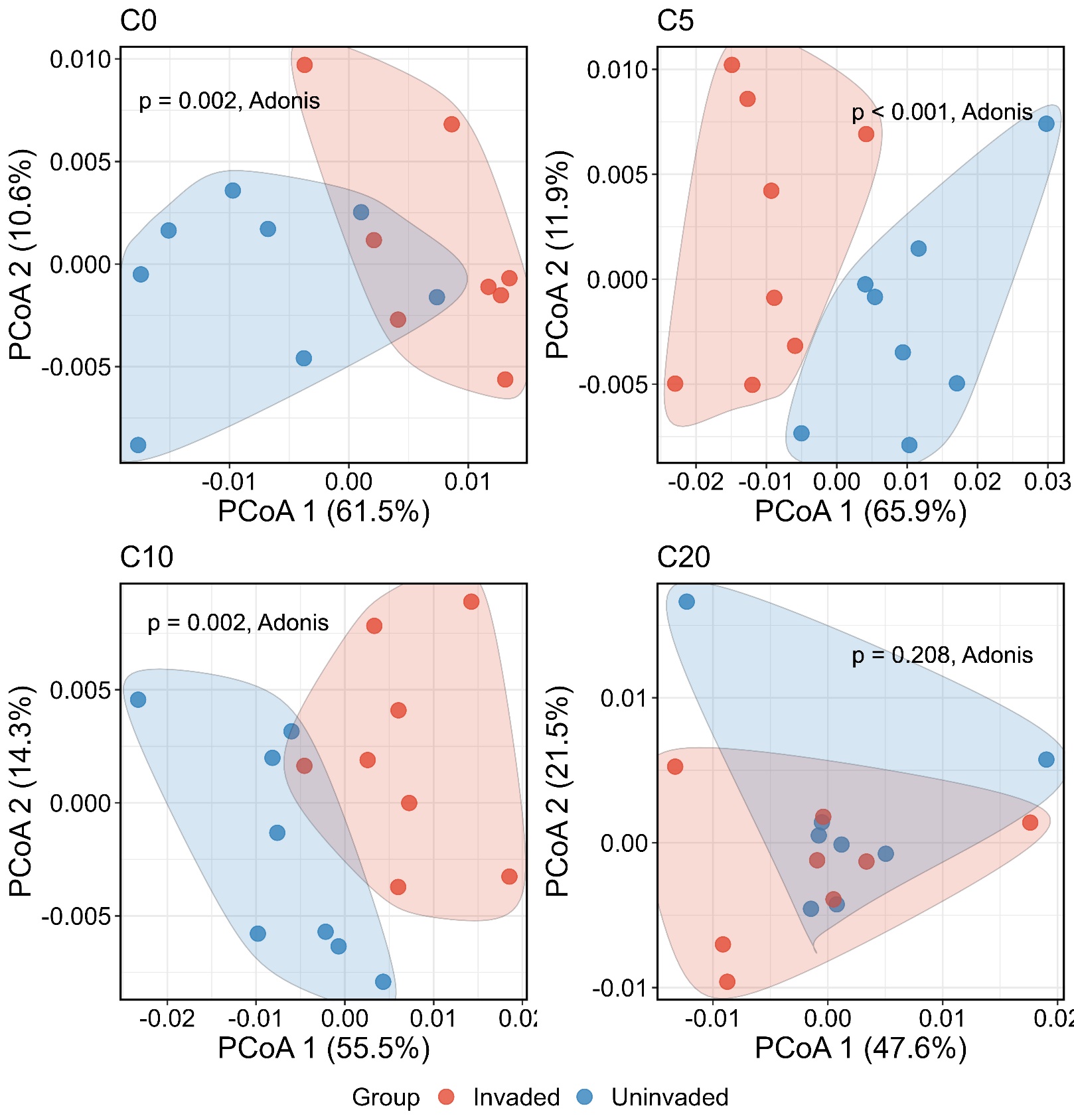


Figure S10. The principal coordinates analysis (PCoA) of community structures after invasion is based on the weighted UniFrac distance. The difference between uninvaded and invaded communities was tested by Adonis.


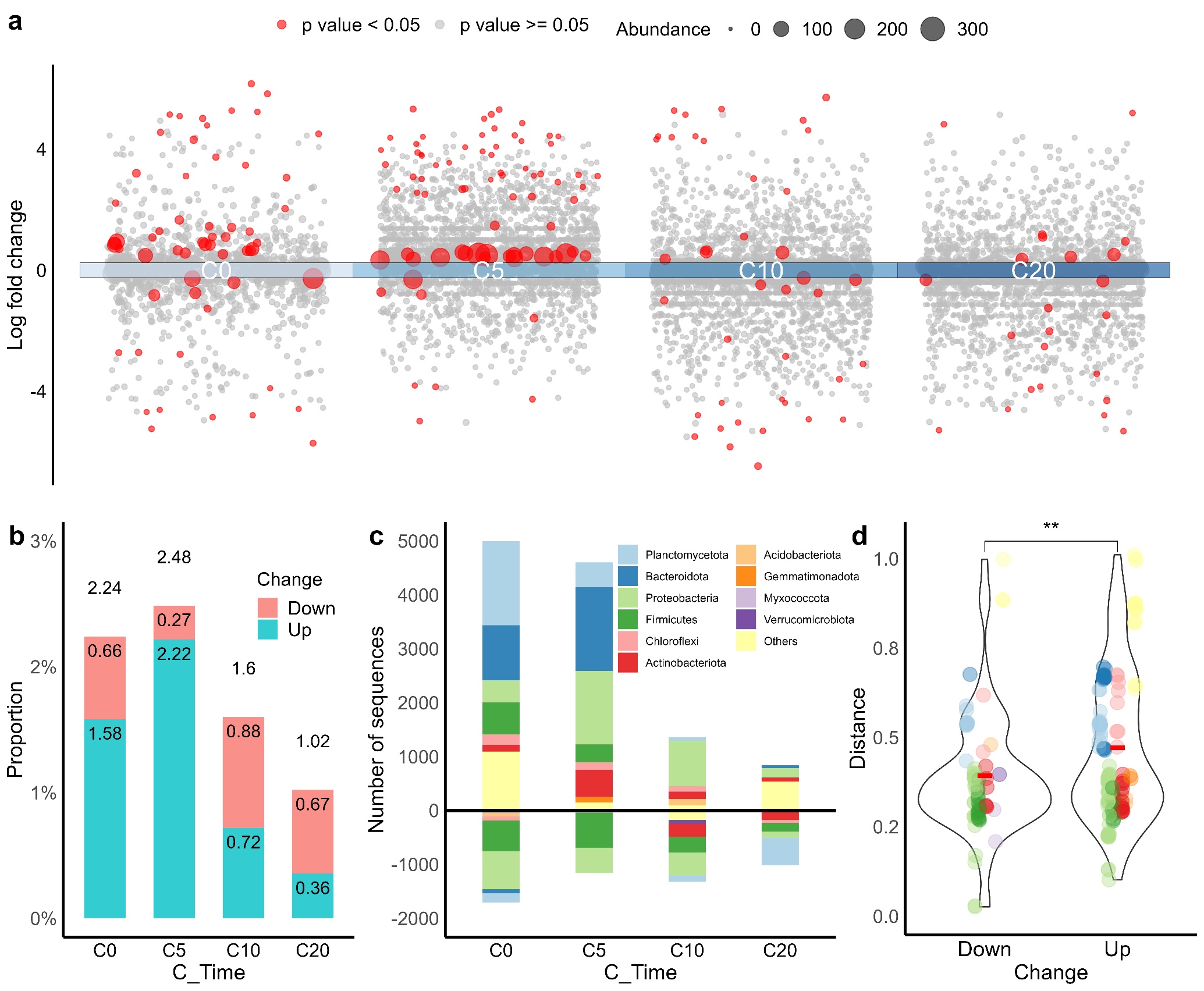


Figure S11. (a) Compared to the uninvaded community, there is a significant increase and decrease (red dots, *p* < 0.05, N=8) of bacterial taxa abundances upon invasion. (b) The proportion (%) of ASVs increased (Up) and decreased (Down) in abundance in each community. (c) Changes of bacterial taxa in the total number of ASVs of each Phylum. (d) The phylogenetic distance between each changed taxa in four communities and four ASVs affiliated with the genus *Escherichia*. The red bars of the two groups indicate the mean values. A significant difference in the mean of such distance was found between increased (Up) and decreased (Down) ASV groups as measured by the t-test (**, *p* < 0.01).


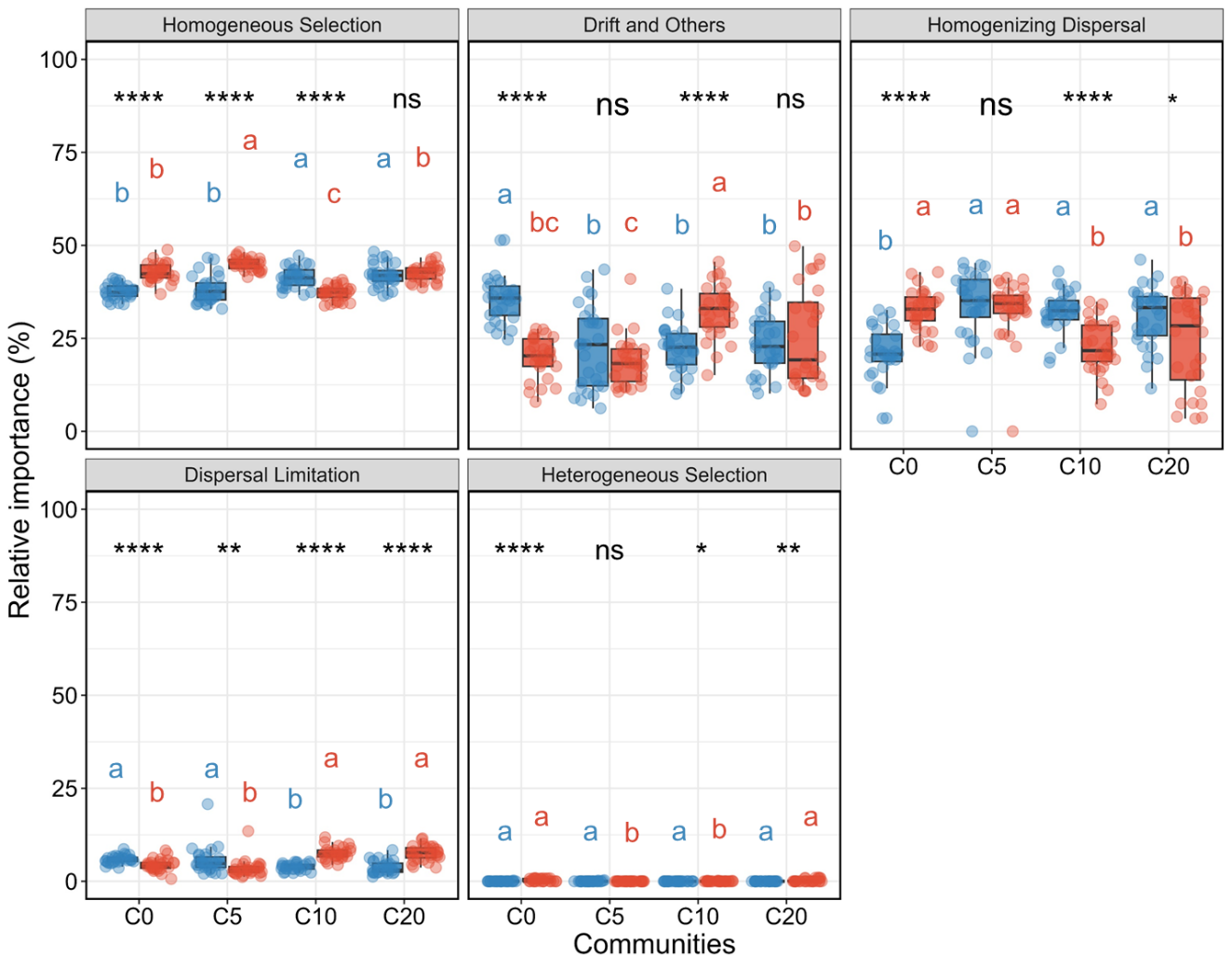


Figure S12. The impact of *E. coli* invasion on community assembly process estimated by iCAMP. The asterisks represent statistical significance between invaded and uninvaded treatments while the letters indicate the difference among communities (ANOVA with post-hoc Tukey HSD test).

Supplementary Tables

Table S1 ASVs affiliated with the genus *Escherichia*.

| ASV_ID | Kingdom | Phylum | Class | Order | Family | Genus |
| --- | --- | --- | --- | --- | --- | --- |
| 73fbcbe902ee1c5189579b0b5c5bc4ce | Bacteria | Proteobacteria | Gammaproteobacteria | Enterobacterales | Enterobacteriaceae | Escherichia |
| 0e24e199833700ff4a38d749878fcf96 | Bacteria | Proteobacteria | Gammaproteobacteria | Enterobacterales | Enterobacteriaceae | Escherichia |
| 60b42be73d780309ad71a4d743cd0503 | Bacteria | Proteobacteria | Gammaproteobacteria | Enterobacterales | Enterobacteriaceae | Escherichia |
| efaf1080ad476bd95cab0a5ee52778e7 | Bacteria | Proteobacteria | Gammaproteobacteria | Enterobacterales | Enterobacteriaceae | Escherichia |

**References**

1. Mawarda PC, Lakke SL, Elsas JD van, Salles JF. Temporal dynamics of the soil bacterial community following Bacillus invasion. *iScience* 2022; **25**: 104185.

2. Gohl DM, Vangay P, Garbe J, MacLean A, Hauge A, Becker A, et al. Systematic improvement of amplicon marker gene methods for increased accuracy in microbiome studies. *Nat Biotechnol* 2016; **34**: 942–949.

3. Li H, Yang S, Semenov MV, Yao F, Ye J, Bu R, et al. Temperature sensitivity of SOM decomposition is linked with a K‐selected microbial community. *Glob Change Biol* 2021; **27**: 2763–2779.

4. Stoddard SF, Smith BJ, Hein R, Roller BRK, Schmidt TM. rrnDB: improved tools for interpreting rRNA gene abundance in bacteria and archaea and a new foundation for future development. *Nucleic Acids Res* 2015; **43**: D593-598.

5. Wu L, Yang Y, Chen S, Jason Shi Z, Zhao M, Zhu Z, et al. Microbial functional trait of rRNA operon copy numbers increases with organic levels in anaerobic digesters. *ISME J* 2017; **11**: 2874–2878.

6. Roller BRK, Stoddard SF, Schmidt TM. Exploiting rRNA operon copy number to investigate bacterial reproductive strategies. *Nat Microbiol* 2016; **1**: 1–7.

7. Graves S, Dorai-Raj H-PP and LS with help from S. multcompView: Visualizations of Paired Comparisons. 2019.

8. Kassambara A. ggpubr: ‘ggplot2’ Based Publication Ready Plots. 2020.

9. Oksanen J, Simpson GL, Blanchet FG, Kindt R, Legendre P, Minchin PR, et al. vegan: Community Ecology Package. 2022.

10. Ning D, Yuan M, Wu L, Zhang Y, Guo X, Zhou X, et al. A quantitative framework reveals ecological drivers of grassland microbial community assembly in response to warming. *Nat Commun* 2020; **11**: 4717.

11. Love MI, Huber W, Anders S. Moderated estimation of fold change and dispersion for RNA-seq data with DESeq2. *Genome Biology* 2014; **15**: 550.

12. Paradis E, Blomberg S, Bolker B, Brown J, Claramunt S, et al. ape: Analyses of Phylogenetics and Evolution. 2022.
